# A new model integrating short- and long-term aging of copper added to soils

**DOI:** 10.1371/journal.pone.0182944

**Published:** 2017-08-18

**Authors:** Saiqi Zeng, Jumei Li, Dongpu Wei, Yibing Ma

**Affiliations:** 1 Institute of Agricultural Resources and Regional Planning, Chinese Academy of Agricultural Sciences, Beijing, P.R. China; 2 School of Resources and Environment, University of Jinan, Jinan, Shandong, P.R. China; RMIT University, AUSTRALIA

## Abstract

Aging refers to the processes by which the bioavailability/toxicity, isotopic exchangeability, and extractability of metals added to soils decline overtime. We studied the characteristics of the aging process in copper (Cu) added to soils and the factors that affect this process. Then we developed a semi-mechanistic model to predict the lability of Cu during the aging process with descriptions of the diffusion process using complementary error function. In the previous studies, two semi-mechanistic models to separately predict short-term and long-term aging of Cu added to soils were developed with individual descriptions of the diffusion process. In the short-term model, the diffusion process was linearly related to the square root of incubation time (t^1/2^), and in the long-term model, the diffusion process was linearly related to the natural logarithm of incubation time (lnt). Both models could predict short-term or long-term aging processes separately, but could not predict the short- and long-term aging processes by one model. By analyzing and combining the two models, we found that the short- and long-term behaviors of the diffusion process could be described adequately using the complementary error function. The effect of temperature on the diffusion process was obtained in this model as well. The model can predict the aging process continuously based on four factors—soil pH, incubation time, soil organic matter content and temperature.

## 1. Introduction

The term “aging” refers to the process by which the bioavailability/toxicity, isotopic exchangeability, and extractability of metals added to soil decline over time. This process is also occasionally referred to as “natural attenuation” or “fixation” [1,2]. After water-soluble copper (Cu) is added to soil, it instantly partitions between solid and solution phases, and the lability of Cu decreases overtime [3–11]. Previous studies have shown that aging can be mainly attributed to three processes: precipitation/nucleation, occlusion within organic matter, and micropore/mesopore diffusion [12–18]. Organic matter content and soil pH value are vital factors that affect occlusion and precipitation/nucleation processes separately, while time and temperature have a strong influence on the diffusion process [4,5,19–24].

Understanding or even predicting aging processes would greatly improve our ability to develop criteria and protocols for ecological risk assessments. Previous studies have developed models to predict the aging processes of metals added to soils [16,25–29]. Ma et al. [27,28] developed two models to predict the lability of Cu in short-term and long-term aging separately using different descriptions of the diffusion process. In the short-term model, the diffusion process was linearly related to the square root of time (t^1/2^ model), and in the long-term model, the diffusion process was linearly related to the natural logarithm of time (lnt model). Since the two models both have simplified descriptions of the diffusion process, they cannot predict the aging process continuously. By analyzing and combining two models [27,28], we found that the short- and long-term behaviors of the diffusion process can be described using the complementary error function, which was described in Crank’s book on diffusion equations [30]. In this study, we developed a new semi-mechanistic model to predict the lability of Cu during aging based on above three known processes, with description of diffusion process using complementary error function. By combining the diffusion equation with the Arrhenius equation, the effect of temperature on the diffusion process was considered as well. The model was validated by testing against 19 soil samples from different European countries with a wide range of physiochemical properties, and the results showed that the model successfully predicted the lability of Cu added to soils.

## 2. Materials and methods

### 2.1 Soil samples and treatments

Nineteen soil samples were collected from European countries with different physiochemical properties. The properties of selected soil samples can be found in the previous papers [27,28]. Briefly, pH values of soil samples ranged from 2.98 to 7.52, clay contents ranged from 5% to 51%, organic carbon contents ranged from 0.41% to 23.32%, CaCO_3_ contents ranged from less than 0.5% to 47.4%, soil total Cu ranged from 1.7 mg/kg to 88 mg/kg, and the concentration of Cu for EC10 treatment (the concentration of Cu that would decrease plant growth by 10%) ranged from 10 mg/kg to 650 mg/kg (soil properties and geological coordination of sample locations are shown in S1 Table). Effective concentrations of Cu (EC10) were added to soil samples. The volume of added Cu was 100 μl with concentrations ranging from 0.2 to 13 g Cu/L. After Cu addition, the short-term samples were mixed, incubated indoors at different constant temperatures, and monitored to maintain moisture levels. The long-term samples were incubated outdoors in Canberra, Australia.

### 2.2 Determination of Cu lability

The lability of Cu (also defined as isotopically exchangeable pool or E value) was determined using a stable (^65^Cu) isotope dilution technique. The procedures and calculation details were provided by Nolan et al. [31]. Briefly, 20 ml Milli-Q water was added to soil samples, then two drops of toluene were added to soil samples to inhibit microbial activities. The soil samples were equilibrated for 24 hours in an end-over-end shaker and then supplemented with carrier-free ^64^Cu (50 μl solution containing ^64^Cu, 60 MBq/ml) or with a small volume of solution (15 mg Cu/kg) containing enriched ^65^Cu equivalent to 0.25 mg Cu/kg for the soils without Cu addition. Soil samples were then returned to the shaker and equilibrated for a further 24 hours. At the end of equilibration, the samples were centrifuged at a relative centrifuge force of 4,000 g for 20 minutes and filtered through 0.2 μm cellulose acetate filters. The E value was calculated as follows:

$$E=R\times \frac{\mathrm{AM}({\mathrm{Cu}}_{\mathrm{nat}})}{\mathrm{AM}\left({}^{65}\mathrm{Cu}\right)}\times \frac{{\mathrm{IR}}_{\mathrm{sp}}-{\mathrm{IR}}_{\mathrm{meas}}}{{\mathrm{IR}}_{\mathrm{meas}}-{\mathrm{IR}}_{\mathrm{mat}}}\times ({\mathrm{IR}}_{\mathrm{nat}}+1)$$ (1)

where R refers to the total concentration of ^65^Cu added to soil (mg/kg soil), AM(Cu_nat_) and AM(^65^Cu) refer to the atomic mass of natural Cu (63.546 amu) and the atomic mass of ^65^Cu (64.928 amu) separately, IR_nat_ is the natural abundance ratio of ^63^Cu/^65^Cu in the soil solution, IR_sp_ is the ratio of Cu in the enriched ^65^Cu solution (^63^Cu*/^65^Cu* or IR_sp_ = 0.5/99.5), and IR_meas_ is the measured Cu isotope ratio (^63^Cu+^63^Cu*/^65^Cu+^65^Cu*) in solution after addition. By subtracting the E value in the control soil (without added Cu) from the E value measured in soil with added Cu, we calculated the E value of added Cu. The E values in the control soils without Cu addition ranged from 0.6 to 14 mg/kg, with a mean value of 3.7 mg/kg. Other information on soil properties and treatments are described in detail in the previous papers [27,28].

### 2.3 Modeling of Cu lability

#### 2.3.1 Lability of Cu added to soils

The lability of added Cu in soil samples was determined using a stable isotope dilution technique [31]. This technique allows a direct assessment of non-isotopically exchangeable Cu. Thus, it is well suited for studying aging processes and modeling. The E value can be expressed by the following equation:

$$E_{\mathrm{add}}=1-Y_{1}-Y_{2}-Y_{3}$$ (2)

where E_add_ represent the lability of added Cu in soils; Y_1_ represents the change (fraction) in E_add_ value fast processes (precipitation/nucleation); Y_2_ represents the change (fraction) in the E_add_ value due to the Cu occlusion within organic matter. Y_3_ represents the change (fraction) in E_add_ value attributed to the diffusion process.

#### 2.3.2 Modeling of precipitation/nucleation processes

When water-soluble Cu is added to soils, it partitions instantly between solid and solution phases, followed by a further, slow process that decreases the lability of added Cu (E_add_). The isotopic exchangeability of added Cu decreases rapidly at the beginning, especially when soil pH value is relatively high (pH>6 in this paper, data in S2 Table). This can be attributed to the precipitation/nucleation process, which is related to the formation of Cu(OH)^+^ in soils [32–34] and can be described by the following equation:

$${{[\mathrm{Cu}(H}_{2}{O)}_{n}]}^{2+}={{[\mathrm{Cu}(\mathrm{OH})(H}_{2}{O)}_{n-x-1}]}^{+}+{\mathrm{xH}}_{2}O+H^{+}\;\left(pK{}^{\circ}=7.7\right)$$ (3)

The reaction above is promoted at high pH values. Although this process needs to be further examined by more direct experiments, such as spectroscopic studies, previous studies on the aging processes of other heavy metal ions, such as Zn, Ni, Co, and Cr, have shown comparable results [10,34–36]. As water molecules more readily dissociate on soil surfaces, this phenomenon will also promote the reaction described above [37]. The chemical equilibrium constant of the equation can be described as:

$$K=\frac{{[H}^{+}{][\mathrm{Cu}(\mathrm{OH})}^{+}]}{{[\mathrm{Cu}}^{2+}]}$$ (4)

According to Eqs (2) and (3), the proportion of Cu(OH)^+^ to total Cu ions (Cu(OH)^+^ and Cu^2+^) can be expressed by the following equation:

$$\frac{{\left[\mathrm{Cu}(\mathrm{OH}\right)}^{+}]}{{\left[\mathrm{Cu}(\mathrm{OH}\right)}^{+}]+{[\mathrm{Cu}}^{2+}]}=\frac{1}{10^{\left(\mathrm{pK}{}^{\circ}-\mathrm{pH}\right)}+1}$$ (5)

To describe the precipitation/nucleation process, Ma et al. [27–28] assumed that the precipitation/nucleation processes are linearly related to the proportion of Cu(OH)^+^ to total Cu ions (Cu(OH)^+^ and Cu^2+^) in solution, and can be described as follows:

$$Y_{1}=\frac{B}{10^{\left(\mathrm{pK}{}^{\circ}\text{-}\mathrm{pH}\right)}+1}\times t^{C/t}$$ (6)

where Y_1_ represents the change (fraction) in E_add_ value due to fast processes (precipitation/nucleation); B is a coefficient that describes the effect of precipitation/nucleation; t is aging time (day); pK° is the first hydrolysis constant of Cu; the equation t^C/t^ (where C is a constant) describes the kinetics of the fast processes; and pH is the soil pH value measured in 0.01M CaCl_2_. As the precipitation/nucleation processes only take a very short time to reach equilibrium, it appears that the fast processes have little reliance on temperature [36], thus the effect of temperature on precipitation/nucleation processes can be ignored. This equation has also been applied to the aging process of other metal ions, such as nickel (Ni) [38] and showed reliable results. Thus, in this paper, we also use this equation to describe the precipitation/nucleation processes.

#### 2.3.3 Modeling of occlusion processes

The amount of Cu occlusion within organic matter is linear related to the amount of organic matter in soils. The process of Cu occlusion within organic matter can be described by the following equation:

$$Y_{2}=F\times (C_{org}/100)\times t^{G/t}$$ (7)

where Y_2_ represents the change (fraction) in the E_add_ value due to Cu occlusion within organic matter; F is a constant that is related to the effect of the occlusion process; C_org_ is total organic carbon content of the soil (%, w/w). The equation t^G/t^ describes the relatively rapid processes of occlusion.

#### 2.2.4 Modeling of diffusion processes

Previous studies have shown that in short-term aging, the dominant process is pH dependent. The precipitation/nucleation processes are dominant in soil samples with high pH values; and diffusion process is dominant while in soil samples with low pH values [4,5,19–24]. In long-term aging, the diffusion process dominates the aging process at all pH conditions as diffusion takes longer time to reach equilibrium, but the precipitation/nucleation process also plays a significant role under high pH conditions. As mentioned previously, Ma et al. [27,28] separately developed two semi-mechanistic models with simplified descriptions of the diffusion process for short-term and long-term aging. Both models showed good results, and are widely used in other studies of the aging process [11,38–40]. However, neither of two models could predict the short- and long-term aging processes continuously.

The kinetics of the diffusion process of Cu ions into mesopores/micropores in soils was studied by monitoring the Cu concentration in solution while the suspension was stirred continuously. As the volume of solution is relatively small compared with the volume of soil, the concentration of solute decreased during the diffusion process. Thus, the diffusion process can be described as “diffusion in a plane sheet from a stirred solution of limited volume” and can be expressed by the following equation based on Crank’s book [30]:

$$\frac{Y_{3}}{1-Y_{1}-Y_{2}}=1-{\exp(T/\alpha }^{2})\times \mathrm{erfc}\sqrt{{T/\alpha }^{2}}$$ (8)

where Y_3_ represents the change (fraction) in E_add_ value attributed to the diffusion process; α is a constant whose value can be given by the fraction of the total amount of Cu finally taken up by soils (f = 1/(1+α), f is the fraction of the amount of Cu taken up by soil to the amount of total Cu added to soil); T = Dt/*l*^2^, where D is the diffusion coefficient, t is incubation time, 2*l* is the sheet thickness; erfc refers to complementary error function.

The relationship between D and temperature follows the Arrhenius equation and can be expressed as follow:

$$D=D_{0}e^{\text{-}\mathrm{Ea/RT}}$$ (9)

Thus, Eq (8) can be expressed as:

$$\frac{Y_{3}}{1-Y_{1}{\text{-}Y}_{2}}=1-{\exp(D}_{0}e^{\text{-}\mathrm{Ea}/\mathrm{RT}}\mathrm{t/}\alpha^{2}l^{2})\times erfc\sqrt{D_{0}e^{\text{-}\mathrm{Ea}/\mathrm{RT}}\mathrm{t/}\alpha^{2}l^{2}}$$ (10)

In the equation above, D_0_, R, α, and E_a_ are constants, thus we can assume that constant N = D_0_/α^2^*l*^2^ and constant K = −E_a_/R. Thus, Eq (10) can be expressed as:

$$\frac{Y_{3}}{{1\text{-}Y}_{2}{\text{-}Y}_{3}}=1-{\exp(\mathrm{Ne}}^{K/T}t)\times erfc\sqrt{{\mathrm{Ne}}^{K/T}t}$$ (11)

By combining Eqs (6), (7), (11), Eq (2) can be expressed as following:

$$E_{\mathrm{add}}={\exp(\mathrm{Ne}}^{\mathrm{K/T}}t)\times \mathrm{erfc}\sqrt{{\mathrm{Ne}}^{K/T}t}\times [1-\frac{B}{10^{{(\mathrm{pK}}^{\circ }-\mathrm{pH})}+1}\times t^{\mathrm{C/t}}-F\times (C_{\mathrm{org}}/100)\times t^{\mathrm{G/t}}]$$ (12)

Regression analysis and data fitting were performed using Microsoft Excel^®^. The parameters in the model were optimized by minimizing the sum of the squares of the residual variation of the data points.

## 3. Results and discussion

### 3.1 Effect of incubation time, soil pH, and temperature

The diffusion process of aging is strongly influenced by incubation time; it also shows a reliance on temperature as well. The precipitation/nucleation and occlusion process are not time dependent, but they are influenced by pH value and organic content separately. The experimental data showed that the lability of Cu generally decreases with incubation time with different decreasing rates of Cu lability at different periods of aging. In initial period of aging, the short-term period, the lability of Cu declines relatively fast, especially in soil samples with relatively high pH value, then followed by a lower decreasing rate in long term aging and finally becomes stable. The influence of soil pH values on the decreasing rate of Cu lability during the short term of aging was due to the occurrence of precipitation/nucleation process in this period. The positive relation between decreasing rate of Cu lability and soil pH value at the short-term aging observed in this and in previous investigations indicates that the dominant processes depend on pH conditions. As stated above in Eq (3), the precipitation/nucleation process is related to the formation of Cu(OH)^+^ in soils [32–34]. Based on the data (S2 Table), we can see that in acidic soil samples, the formation of Cu(OH)^+^ was inhibited and precipitation/nucleation process plays an minor role in aging process, thus the diffusion process dominates aging initially. While in neutral and alkali soil samples, the formation of Cu(OH)^+^ was supported as water molecules are more likely to dissociate than in acidic soil samples, and the precipitation/nucleation process dominates the early stage of aging [28,41]. Donner et al. [36] found that very rapid reactions (<15 s) decreased the lability of Zn, and the brevity of reaction time excluded the possibility of simple sorption or a diffusion process that is typically attributed to the fast reactions.

**Fig 1:**
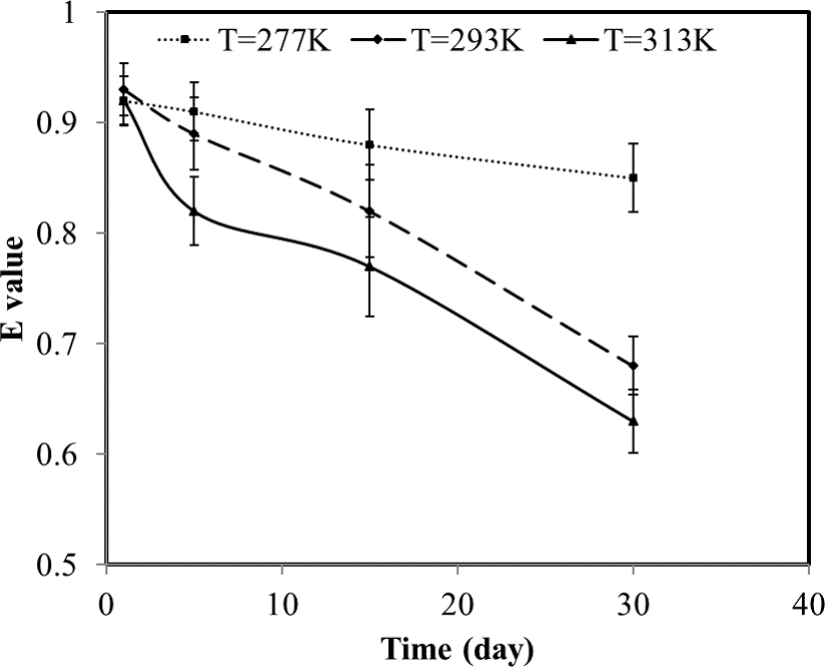
Average Cu labile pool (E value as fraction of total added Cu) in 17 short-term soil samples as a function of incubation time and temperature (vertical lines represent the standard errors).

Short-term data showed that the decreasing rate of Cu lability is positively related to temperature as well (Fig 1). That is probably because Cu ions have higher free energy at higher temperatures, thus processes attributed to aging are enhanced, especially the diffusion process.

### 3.2 Determination and analysis of parameters in erfc model

The model we developed in this paper (the erfc model) based on nucleation/precipitation, mesopore/micropore diffusion, and occlusion within organic matter was used to describe the aging process that leads to the decrease in isotopic exchangeability of Cu (lability of E value) added to soils.

To keep fewer variables in the model, the value of parameters K and pK° can be determined based on previous papers. In the previous paper [27] about short-term aging of Cu, the mean value of estimated activation energy was 36 KJ/mol. Thus, the parameter K = −E_a_/R = -4330. Also, the value of the first hydrolysis constant of Cu in bulk solution (pK°) was fixed at 7.7 in accordance with the value reported by Lindsay [42] and Wolt [43]. If the value of pK° was estimated in this model, there was little change in the coefficient of determination (R^2^) and root-mean-square error (RMSE). The estimated value of pK° was approximately 6.65, lower than the first hydrolysis constant of Cu in bulk solution (7.7). This result agreed with the results of a previous study that determined the hydrolysis of Cu ions in soils was promoted by soil solid surfaces [37,44,45]. In this paper, the first hydrolysis constant of Cu was fixed at 7.7 to keep fewer variables in the model.

As the incubation time of the data ranges from 0 to 360 days in this paper, there might be some uncertainty to predict the E_add_ value when incubation time is longer than decades. To further improve the model, it is necessary to control the trend of the model when incubation time is relatively long. In the previous paper about long-term aging of Cu [28], the lnt model had predicted the aging process of Cu with long incubation time successfully (range from 8 to 78 years). Thus, we use the predicted E values of 3600 and 7200 days of lnt model as the conditions to control the trend of the erfc model in this paper.

Regression analysis and data fitting were performed using the Solver Function of Microsoft Excel^®^. The parameters in the model were optimized by minimizing the sum of the square of the residual variation of the data points from the model. The parameters were subjected to the constraints: B≥0, N≥0, C≥0, F≥0, G≥0, with 0.00001 precision, 5% tolerance and 0.001 convergences.

By analyzing the previous model developed by Ma et al. [28] and expression of Y_2_ in this paper, we found that the value of Y_2_ in erfc model was almost linearly related to temperature when it ranged from 253K to 323K. Thus, we could use the average temperature of the aging period before the E value was measured to predict the aging processes.

**Fig 2:**
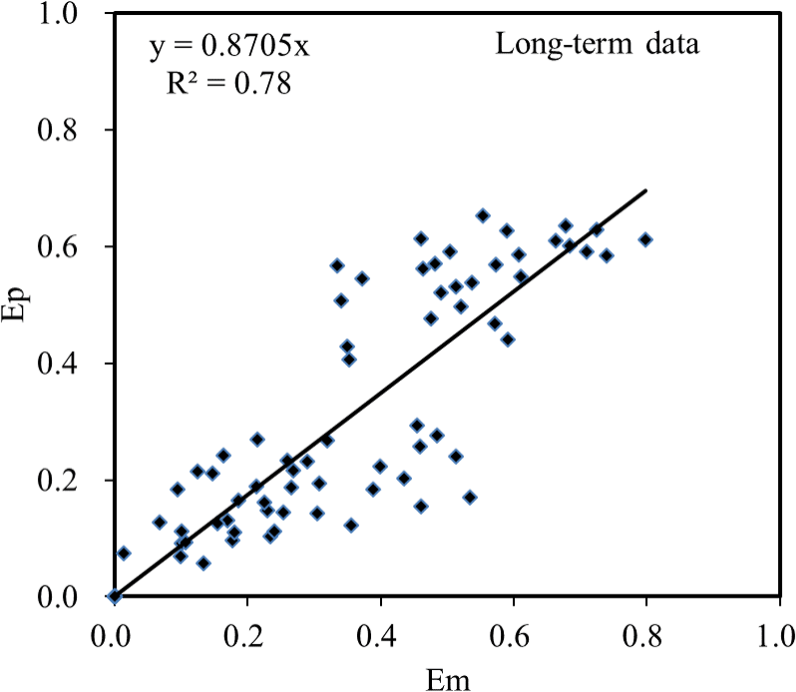
The measured E values (E_m_) versus the predicted E values of the erfc model (E_p_).

The estimated values of other parameters, R^2^ and RMSE are shown in Table 1. The relation of the measured E value (E_m_) versus the predicted E value by the erfc model (E_p_) are shown in Fig 2.

**Table 1:** The estimated values of parameters, R^2^ and RMSE in erfc model.

B	C	N	F	G	R^2^	RMSE
1.14	0	214.91	2.85	0	0.789	0.113

The estimated value of parameter C is zero, thus, equations t^C/t^ = 1. This indicates that precipitation/nucleation processes take a very short time and can be considered to be stable with time comparing with whole aging period, which keeps accordance with Donner’s previous result [36]. G = 0 shows the similar result that occlusion has little time reliance as well. The erfc model can be expressed as follows:

$$E_{\mathrm{add}}=\exp(214{.91e}^{\text{-}4330/T}t)\times \mathrm{erfc}\sqrt{214{.91e}^{\text{-}4330/T}t}\times [1-\frac{1.14}{10^{\left(7.7-\mathrm{pH}\right)}+1}-2.85\times (C_{\mathrm{org}}/100)]$$ (13)

Fig 2 shows the measured E values (E_m_) versus the predicted E values by the semi-mechanistic model (E_p_) for the long-term data. It should be noted that there are some errors in E_m_ and E_p_ besides the experimental variations. For example, the soil samples were incubated outdoor for long time and no specific treatments were deployed to inhibit microbial activities, thus some microbial activities might affect the lability of Cu. Also, the water content of soil, which would influence the diffusion and precipitation/nucleation processes, was strongly depended on weather conditions and not considered in erfc model. This would cause some variations between E_m_ and E_p_ values. Moreover, the temperatures we used in erfc model were the average temperatures during the incubation periods in Canberra, which might different from actual temperature in specific times. Despite these factors that may cause some variations between E_m_ and E_p_, the erfc model we developed in this paper still showed a satisfactory result.

### 3.3 Validation of the model

**Fig 3:**
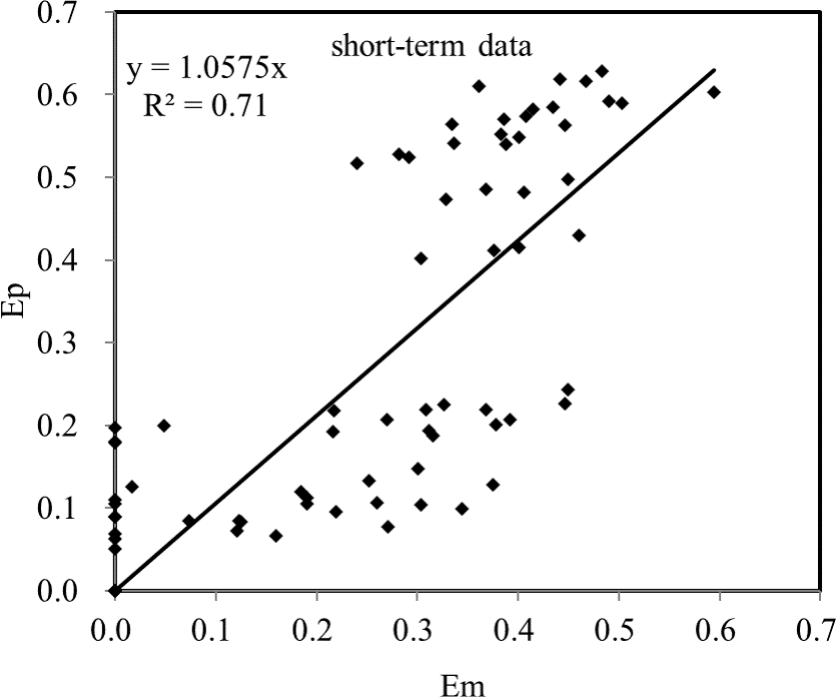
The measured E values (E_m_) versus the E values predicted by erfc model (E_p_) for short-term data.

The measured E values of short-term soil samples (15 and 30 days, at temperature 293K and 313K) in previous work [27] were in good agreement with those predicted by erfc model (Fig 3).

The short-term samples were incubated indoors under controlled experimental conditions that would affect aging, such as temperature, moisture, and microbial activities; while in the long-term aging experiment, soil samples were incubated outdoors in Canberra. As the parameters in the model were estimated using long-term data, the different experimental conditions between short- and long-term aging experiments are responsible for the variations shown in Fig 3. Also, a short time at ambient temperature before the E value was measured after sampling attributed to variations between E_m_ and E_p_ as well. However, the erfc model developed in this study still showed good results when tested against short-term data. The model showed reliable results in predicting the lability of Cu in both short-term and long-term aging despite some tolerable variations caused by the divergences of experimental conditions between the two experiments.

**Fig 4:**
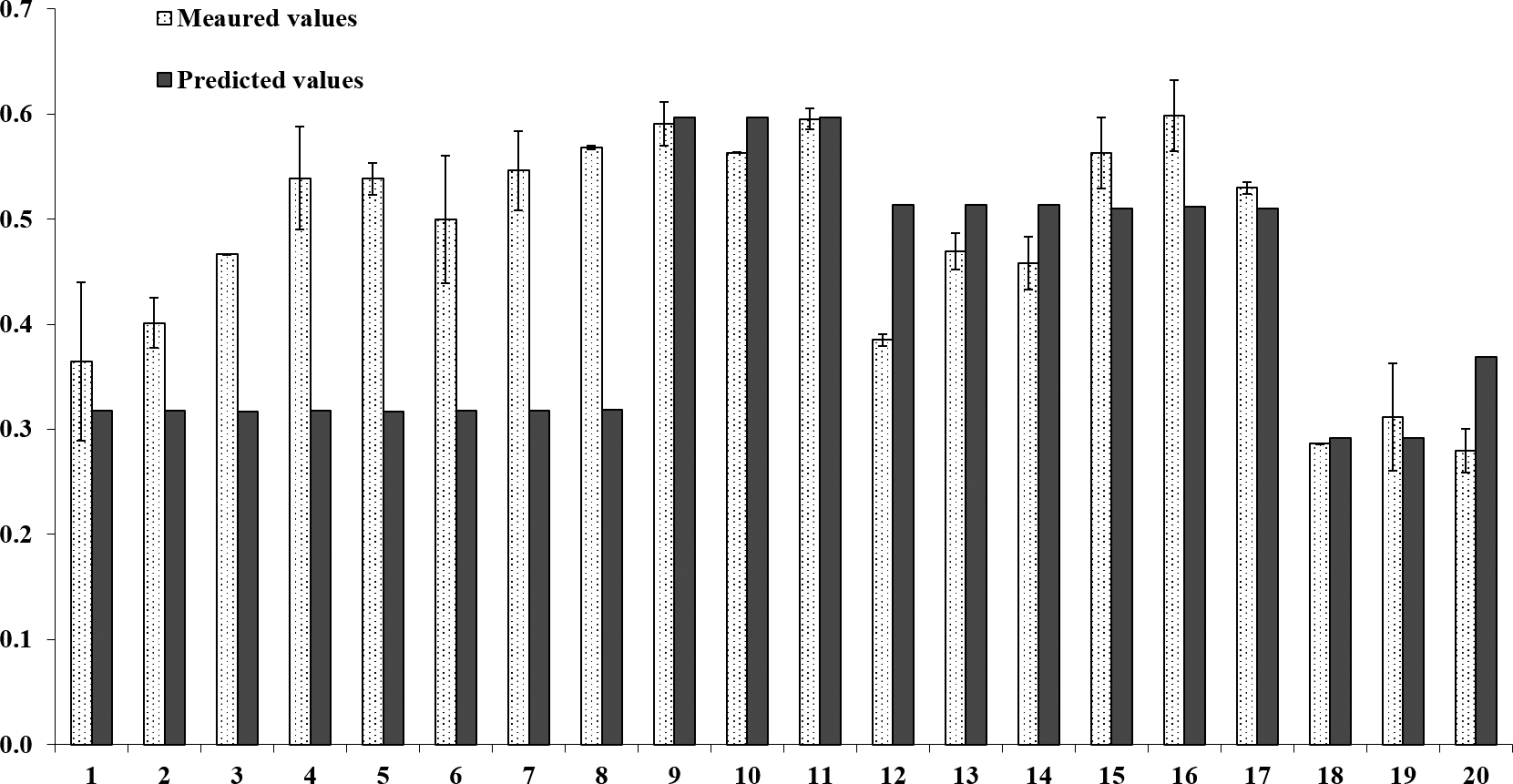
The measured E values and predicted E values of field-contamination soil samples. Vertical lines represent standard errors where they exceed the height of columns.

The erfc model is further validated by testing it against 20 field-contaminated/incubated soil samples. The properties of soil samples were described in database and previous papers [8,28,46]. Briefly, the properties are shown in Table 2, the temperatures are the annual average temperatures of the location. The measured E values and predicted E values of field-contamination soils are shown in Fig 4. As the soil samples in erfc model were treated with total Cu concentration that would decrease plant growth by 10% (EC10 treatment), in some soil samples with high total Cu concentration, such as Hygum3-Hygum8, the precision of erfc model may be affected.

**Table 2:** Soil pH, temperature (K), time (year, since soil field-contamination with Cu salts), soil organic carbon content (w/w%), total Cu (mg kg^-1^), measured E values (E_m_, fraction).

No.	Location	Coordinates	Soil pH	Temperature	Time	SOC	Total Cu	E_m_
1	Hygum1	55°46’30”N, 9°25’43”E	5.43	288.0	78	2.58	41.1	0.36
2	Hygum2	55°46’30”N, 9°25’43”E	5.53	288.0	78	2.58	75.3	0.40
3	Hygum3	55°46’30”N, 9°25’43”E	5.63	288.0	78	2.58	201.1	0.47
4	Hygum4	55°46’30”N, 9°25’43”E	5.49	288.0	78	2.58	298.2	0.54
5	Hygum5	55°46’30”N, 9°25’43”E	5.62	288.0	78	2.58	304.5	0.54
6	Hygum6	55°46’30”N, 9°25’43”E	5.48	288.0	78	2.58	337.1	0.50
7	Hygum7	55°46’30”N, 9°25’43”E	5.42	288.0	78	2.58	406.4	0.55
8	Hygum8	55°46’30”N, 9°25’43”E	5.19	288.0	78	2.58	463.6	0.57
9	Woburn1	51°59’17”N, 0°35’30”W	6.36	282.5	8	1.5	45.6	0.59
10	Woburn2	51°59’17”N, 0°35’30”W	6.36	282.5	8	1.5	89.8	0.56
11	Woburn3	51°59’17”N, 0°35’30”W	6.36	282.5	8	1.5	115.6	0.60
12	WagningenA1	51°57’49”N, 5°39’54”E	3.86	282.5	22	1.5	27.5	0.38
13	WagningenA2	51°57’49”N, 5°39’54”E	3.87	282.5	22	1.5	45.4	0.47
14	WagningenA3	51°57’49”N, 5°39’54”E	3.98	282.5	22	1.5	44.9	0.46
15	WagningenD1	51°57’49”N, 5°39’54”E	5.43	282.5	22	1.5	46.0	0.56
16	WagningenD2	51°57’49”N, 5°39’54”E	5.14	282.5	22	1.5	55.5	0.60
17	WagningenD3	51°57’49”N, 5°39’54”E	5.48	282.5	22	1.5	71.2	0.53
18	Italy1	44°41’55”N, 10°37’48”E	7.14	288.5	40	3.1	60.2	0.29
19	Italy2	44°41’55”N, 10°37’48”E	7.14	288.5	40	3.1	121.8	0.31
20	Hungary1	46°54’25” N, 18°31’22” E	7.30	283.5	13	2.7	40.5	0.28

The erfc model is based on the three main processes of aging: precipitation/nucleation, diffusion and occlusion within organic matter. And four vital factors that would influence the lability of Cu were also considered: time, soil pH, temperature and organic carbon content. However, other factors that may have impacts on lability of Cu, such as moisture, microbial activities and plant absorption, were not included in this model. Though the erfc model showed satisfactory results, it can be further improved as future research on the mechanisms of aging provide more detailed information on reactions during the aging process. Moreover, this model can only be applied to water-soluble Cu added to soils, as the lability of other formations of added Cu, such as sewage and sludge or organic fertilizers, cannot be predicted by this model due to different reactions that are attributed to aging in different formations of added Cu.

## 4. Conclusion

When water-soluble Cu is added to soil, the lability of Cu decreases rapidly in the initial period, and this is followed by further decreases but at a slower rate. The lability of Cu added to soils generally decreases with incubation time. The fast reactions are attributed to precipitation/nucleation processes and occlusion within organic matter, soil pH and organic matter content would affect these processes separately. Slow reactions are processes such as diffusion of Cu ions into micropores/mesopores on soil surfaces. Temperature is an important factor that affects the slow processes.

By analyzing and improving a previous model developed by Ma et al. [27,28] on aging of Cu added to soils, we developed a new semi-mechanistic model that can predict the lability of water-soluble Cu added to soil, integrating short-term and long-term aging simultaneously with the description of the diffusion process using the complementary error function. The effect of incubation time, temperature, soil organic matter content and soil pH were considered. The model showed good predicting ability by testing it against short-term and long-term data from a previous study conducted by Ma et al. [27,28]. However, this model needs further improvement through consideration of other factors that affect the aging process, such as moisture, plant absorption, and microbial activities.

## Supporting information

S1 Table Long-term data for modeling. (DOCX)

S2 Table Short-term data for modeling. (DOCX)
