# Supplementary material for: A new model integrating short- and long-term aging of copper added to soils
Source: PLoS One. 2017 Aug 18;12(8):e0182944. doi: 10.1371/journal.pone.0182944 (PMC5562308; doi:10.1371/journal.pone.0182944)
Supplement: S1 Table — (DOCX) [file pone.0182944.s001.docx]

S1 Table. Long-term Data for Modeling

| Soil No | Location | Geological coordination | E (%)-aged | pH(CaCl_2_) | Temperature (K) | Time (day) | Total C (%) |
| --- | --- | --- | --- | --- | --- | --- | --- |
| 1 | Gudow (Germany) | 53°33’22’’N, 10°47’00’’E | 0.00 | 2.98 | 293.00 | 0 | 5.12 |
| 2 | Nottingham (UK) | 52°57’14’’N, 1°10’12’’W | 0.00 | 3.36 | 293.00 | 0 | 5.20 |
| 3 | Houthalen(Belgium) | 51°03’08’’N, 5°25’44’’E | 0.00 | 3.38 | 293.00 | 0 | 1.86 |
| 7 | Kövlinge I (Sweden) | 55°48’49’’N, 13°03’05’’E | 0.00 | 4.76 | 293.00 | 0 | 1.63 |
| 8 | Souli (Greece) | 39°28’17’’N, 20°31’08’’E | 0.00 | 4.80 | 293.00 | 0 | 0.41 |
| 9 | Kövlinge II (Sweden) | 55°48’49’’N, 13°03’05’’E | 0.00 | 5.06 | 293.00 | 0 | 2.35 |
| 10 | Montpellier (France) | 43°36’36’’N, 3°52’26’’E | 0.00 | 5.18 | 293.00 | 0 | 0.76 |
| 12 | Aluminusa (Italy) | 37°51’47’’N, 13°47’15’’E | 0.00 | 5.44 | 293.00 | 0 | 0.87 |
| 14 | Woburn (UK) | 51°59’17’’N, 0°35’30’’W | 0.00 | 6.36 | 293.00 | 0 | 4.40 |
| 15 | Ter Munck (Belgium) | 50°52’42’’ N, 4°39’24’’ E | 0.00 | 6.80 | 293.00 | 0 | 0.98 |
| 16 | Vault de Lugny (France) | 47°30’01’’N, 3°50’38’’E | 0.00 | 7.29 | 293.00 | 0 | 2.19 |
| 17 | Rots (France) | 49°11’33’’N, 0°32’34’’E | 0.00 | 7.38 | 293.00 | 0 | 3.04 |
| 18 | Souli (Greece) | 39°28’17’’N, 20°31’08’’E | 0.00 | 7.38 | 293.00 | 0 | 8.05 |
| 19 | Marknesse (Netherlands) | 52°42’15’’N, 5°53’16’’E | 0.00 | 7.52 | 293.00 | 0 | 2.47 |
| 20 | Barcelona (Spain) | 41°23’41’’N, 2°08’55’’E | 0.00 | 7.49 | 293.00 | 0 | 2.34 |
| 21 | Brécy (France) | 47°07’44’’N, 2°37’53’’E | 0.00 | 7.51 | 293.00 | 0 | 3.63 |
| 22 | Guadalajara (Spain) | 40°36’26’’N, 3°14’12’’E | 0.00 | 7.52 | 293.00 | 0 | 4.00 |
| 1 | Gudow (Germany) | 53°33’22’’N, 10°47’00’’E | 0.09 | 2.98 | 293.00 | 21 | 5.12 |
| 2 | Nottingham (UK) | 52°57’14’’N, 1°10’12’’W | 0.27 | 3.36 | 293.00 | 21 | 5.20 |
| 3 | Houthalen (Belgium) | 51°03’08’’N, 5°25’44’’E | 0.18 | 3.38 | 293.00 | 21 | 1.86 |
| 7 | Kövlinge I (Sweden) | 55°48’49’’N, 13°03’05’’E | 0.10 | 4.76 | 293.00 | 21 | 1.63 |
| 8 | Souli (Greece) | 39°28’17’’N, 20°31’08’’E | 0.13 | 4.80 | 293.00 | 21 | 0.41 |
| 9 | Kövlinge II (Sweden) | 55°48’49’’N, 13°03’05’’E | 0.10 | 5.06 | 293.00 | 21 | 2.35 |
| 10 | Montpellier (France) | 43°36’36’’N, 3°52’26’’E | 0.10 | 5.18 | 293.00 | 21 | 0.76 |
| 12 | Aluminusa (Italy) | 37°51’47’’N, 13°47’15’’E | 0.01 | 5.44 | 293.00 | 21 | 0.87 |
| 14 | Woburn (UK) | 51°59’17’’N, 0°35’30’’W | 0.15 | 6.36 | 293.00 | 21 | 4.40 |
| 15 | Ter Munck (Belgium) | 50°52’42’’ N, 4°39’24’’ E | 0.31 | 6.80 | 293.00 | 21 | 0.98 |
| 16 | Vault de Lugny (France) | 47°30’01’’N, 3°50’38’’E | 0.35 | 7.29 | 293.00 | 21 | 2.19 |
| 17 | Rots (France) | 49°11’33’’N, 0°32’34’’E | 0.47 | 7.38 | 293.00 | 21 | 3.04 |
| 18 | Souli (Greece) | 39°28’17’’N, 20°31’08’’E | 0.46 | 7.38 | 293.00 | 21 | 8.05 |
| 19 | Marknesse (Netherlands) | 52°42’15’’N, 5°53’16’’E | 0.37 | 7.52 | 293.00 | 21 | 2.47 |
| 20 | Barcelona (Spain) | 41°23’41’’N, 2°08’55’’E | 0.49 | 7.49 | 293.00 | 21 | 2.34 |
| 21 | Brécy (France) | 47°07’44’’N, 2°37’53’’E | 0.33 | 7.51 | 293.00 | 21 | 3.63 |
| 22 | Guadalajara (Spain) | 40°36’26’’N, 3°14’12’’E | 0.61 | 7.52 | 293.00 | 21 | 4.00 |
| 1 | Gudow (Germany) | 53°33’22’’N, 10°47’00’’E | 0.12 | 2.98 | 288.63 | 90 | 5.12 |
| 2 | Nottingham (UK) | 52°57’14’’N, 1°10’12’’W | 0.27 | 3.36 | 288.63 | 90 | 5.20 |
| 3 | Houthalen (Belgium) | 51°03’08’’N, 5°25’44’’E | 0.17 | 3.38 | 288.63 | 90 | 1.86 |
| 7 | Kövlinge I (Sweden) | 55°48’49’’N, 13°03’05’’E | 0.15 | 4.76 | 288.63 | 90 | 1.63 |
| 8 | Souli (Greece) | 39°28’17’’N, 20°31’08’’E | 0.11 | 4.80 | 288.63 | 90 | 0.41 |
| 9 | Kövlinge II (Sweden) | 55°48’49’’N, 13°03’05’’E | 0.25 | 5.06 | 288.63 | 90 | 2.35 |
| 10 | Montpellier (France) | 43°36’36’’N, 3°52’26’’E | 0.23 | 5.18 | 288.63 | 90 | 0.76 |
| 12 | Aluminusa (Italy) | 37°51’47’’N, 13°47’15’’E | 0.18 | 5.44 | 288.63 | 90 | 0.87 |
| 14 | Woburn (UK) | 51°59’17’’N, 0°35’30’’W | 0.16 | 6.36 | 288.63 | 90 | 4.40 |
| 15 | Ter Munck (Belgium) | 50°52’42’’ N, 4°39’24’’ E | 0.40 | 6.80 | 288.63 | 90 | 0.98 |
| 16 | Vault de Lugny (France) | 47°30’01’’N, 3°50’38’’E | 0.35 | 7.29 | 288.63 | 90 | 2.19 |
| 17 | Rots (France) | 49°11’33’’N, 0°32’34’’E | 0.52 | 7.38 | 288.63 | 90 | 3.04 |
| 18 | Souli (Greece) | 39°28’17’’N, 20°31’08’’E | 0.59 | 7.38 | 288.63 | 90 | 8.05 |
| 19 | Marknesse (Netherlands) | 52°42’15’’N, 5°53’16’’E | 0.46 | 7.52 | 288.63 | 90 | 2.47 |
| 20 | Barcelona (Spain) | 41°23’41’’N, 2°08’55’’E | 0.54 | 7.49 | 288.63 | 90 | 2.34 |
| 21 | Brécy (France) | 47°07’44’’N, 2°37’53’’E | 0.74 | 7.51 | 288.63 | 90 | 3.63 |
| 22 | Guadalajara (Spain) | 40°36’26’’N, 3°14’12’’E | 0.68 | 7.52 | 288.63 | 90 | 4.00 |
| 1 | Gudow (Germany) | 53°33’22’’N, 10°47’00’’E | 0.29 | 2.98 | 284.00 | 180 | 5.12 |
| 2 | Nottingham (UK) | 52°57’14’’N, 1°10’12’’W | 0.26 | 3.36 | 284.00 | 180 | 5.20 |
| 3 | Houthalen (Belgium) | 51°03’08’’N, 5°25’44’’E | 0.23 | 3.38 | 284.00 | 180 | 1.86 |
| 7 | Kövlinge I (Sweden) | 55°48’49’’N, 13°03’05’’E | 0.31 | 4.76 | 284.00 | 180 | 1.63 |
| 8 | Souli (Greece) | 39°28’17’’N, 20°31’08’’E | 0.24 | 4.80 | 284.00 | 180 | 0.41 |
| 9 | Kövlinge II (Sweden) | 55°48’49’’N, 13°03’05’’E | 0.22 | 5.06 | 284.00 | 180 | 2.35 |
| 10 | Montpellier (France) | 43°36’36’’N, 3°52’26’’E | 0.36 | 5.18 | 284.00 | 180 | 0.76 |
| 12 | Aluminusa (Italy) | 37°51’47’’N, 13°47’15’’E | 0.07 | 5.44 | 284.00 | 180 | 0.87 |
| 14 | Woburn (UK) | 51°59’17’’N, 0°35’30’’W | 0.46 | 6.36 | 284.00 | 180 | 4.40 |
| 15 | Ter Munck (Belgium) | 50°52’42’’ N, 4°39’24’’ E | 0.51 | 6.80 | 284.00 | 180 | 0.98 |
| 16 | Vault de Lugny (France) | 47°30’01’’N, 3°50’38’’E | 0.59 | 7.29 | 284.00 | 180 | 2.19 |
| 17 | Rots (France) | 49°11’33’’N, 0°32’34’’E | 0.34 | 7.38 | 284.00 | 180 | 3.04 |
| 18 | Souli (Greece) | 39°28’17’’N, 20°31’08’’E | 0.68 | 7.38 | 284.00 | 180 | 8.05 |
| 19 | Marknesse (Netherlands) | 52°42’15’’N, 5°53’16’’E | 0.48 | 7.52 | 284.00 | 180 | 2.47 |
| 20 | Barcelona (Spain) | 41°23’41’’N, 2°08’55’’E | 0.61 | 7.49 | 284.00 | 180 | 2.34 |
| 21 | Brécy (France) | 47°07’44’’N, 2°37’53’’E | 0.71 | 7.51 | 284.00 | 180 | 3.63 |
| 22 | Guadalajara (Spain) | 40°36’26’’N, 3°14’12’’E | 0.66 | 7.52 | 284.00 | 180 | 4.00 |
| 1 | Gudow (Germany) | 53°33’22’’N, 10°47’00’’E | 0.32 | 2.98 | 286.00 | 360 | 5.12 |
| 2 | Nottingham (UK) | 52°57’14’’N, 1°10’12’’W | 0.22 | 3.36 | 286.00 | 360 | 5.20 |
| 3 | Houthalen (Belgium) | 51°03’08’’N, 5°25’44’’E | 0.21 | 3.38 | 286.00 | 360 | 1.86 |
| 7 | Kövlinge I (Sweden) | 55°48’49’’N, 13°03’05’’E | 0.39 | 4.76 | 286.00 | 360 | 1.63 |
| 8 | Souli (Greece) | 39°28’17’’N, 20°31’08’’E | 0.46 | 4.80 | 286.00 | 360 | 0.41 |
| 9 | Kövlinge II (Sweden) | 55°48’49’’N, 13°03’05’’E | 0.43 | 5.06 | 286.00 | 360 | 2.35 |
| 10 | Montpellier (France) | 43°36’36’’N, 3°52’26’’E | 0.19 | 5.18 | 286.00 | 360 | 0.76 |
| 12 | Aluminusa (Italy) | 37°51’47’’N, 13°47’15’’E | 0.53 | 5.44 | 286.00 | 360 | 0.87 |
| 14 | Woburn (UK) | 51°59’17’’N, 0°35’30’’W | 0.45 | 6.36 | 286.00 | 360 | 4.40 |
| 15 | Ter Munck (Belgium) | 50°52’42’’ N, 4°39’24’’ E | 0.48 | 6.80 | 286.00 | 360 | 0.98 |
| 16 | Vault de Lugny (France) | 47°30’01’’N, 3°50’38’’E | 0.57 | 7.29 | 286.00 | 360 | 2.19 |
| 17 | Rots (France) | 49°11’33’’N, 0°32’34’’E | 0.51 | 7.38 | 286.00 | 360 | 3.04 |
| 18 | Souli (Greece) | 39°28’17’’N, 20°31’08’’E | 0.55 | 7.38 | 286.00 | 360 | 8.05 |
| 19 | Marknesse (Netherlands) | 52°42’15’’N, 5°53’16’’E | 0.50 | 7.52 | 286.00 | 360 | 2.47 |
| 20 | Barcelona (Spain) | 41°23’41’’N, 2°08’55’’E | 0.57 | 7.49 | 286.00 | 360 | 2.34 |
| 21 | Brécy (France) | 47°07’44’’N, 2°37’53’’E | 0.80 | 7.51 | 286.00 | 360 | 3.63 |
| 22 | Guadalajara (Spain) | 40°36’26’’N, 3°14’12’’E | 0.72 | 7.52 | 286.00 | 360 | 4.00 |
